# Novel non-canonical role of STAT1 in Natural Killer cell cytotoxicity

**DOI:** 10.1080/2162402X.2016.1186314

**Published:** 2016-05-19

**Authors:** Eva Maria Putz, Andrea Majoros, Dagmar Gotthardt, Michaela Prchal-Murphy, Eva Maria Zebedin-Brandl, Daniela Alexandra Fux, Andreas Schlattl, Robert D. Schreiber, Sebastian Carotta, Mathias Müller, Christopher Gerner, Thomas Decker, Veronika Sexl

**Affiliations:** a Institute of Pharmacology and Toxicology, Department for Biomedical Sciences, University of Veterinary Medicine Vienna, Vienna, Austria; b Max F. Perutz Laboratories, University of Vienna, Vienna, Austria; c Institute of Pharmacology, Center for Physiology and Pharmacology, Medical University of Vienna, Vienna, Austria; d Boehringer Ingelheim RCV GmBH & CO KG, Vienna, Austria; e Department of Pathology and Immunology, Washington University School of Medicine, St Louis, MO, USA; f Division of Molecular Immunology, Walter and Eliza Hall Institute of Medical Research, Melbourne, Victoria, Australia; g Department of Medical Biology, The University of Melbourne, Melbourne, Victoria, Australia; h Institute of Animal Breeding and Genetics, University of Veterinary Medicine Vienna, Vienna, Austria; i Biomodels Austria, Vienna, Austria; j Department of Analytical Chemistry, Faculty of Chemistry, University of Vienna, Vienna, Austria

**Keywords:** Interactome; NK cell, STAT1, tumor surveillance, vesicle

## Abstract

STAT1 is an important regulator of NK cell maturation and cytotoxicity. Although the consequences of *Stat1*-deficiency have been described in detail the underlying molecular functions of STAT1 in NK cells are only partially understood. Here, we describe a novel non-canonical role of STAT1 that was unmasked in NK cells expressing a *Stat1-Y701F* mutant. This mutation prevents JAK-dependent phosphorylation, subsequent nuclear translocation and cytokine-induced transcriptional activity as verified by RNA-seq analysis. As expected *Stat1-Y701F* mice displayed impaired NK cell maturation comparable to *Stat1*^*−/−*^ animals. In contrast *Stat1-Y701F* NK cells exerted a significantly enhanced cytotoxicity *in vitro* and *in vivo* compared to *Stat1*^*−/−*^ NK cells in the absence of detectable transcriptional activity. We thus investigated the STAT1 interactome using primary NK cells derived from *Stat1*^*ind*^ mice that inducibly express a FLAG-tagged STAT1. Mass spectrometry revealed that STAT1 directly binds proteins involved in cell junction formation and proteins associated to membrane or membrane-bound vesicles. In line, immunofluorescence studies uncovered the recruitment of STAT1 to the target-cell interphase during NK cell killing. This led us to propose a novel function for STAT1 at the immunological synapse in NK cells regulating tumor surveillance and cytotoxicity.

## Introduction

The signal transducer and activator of transcription 1 (STAT1) protein drives transcriptional programs induced by various cytokines such as interferons (IFNs) and IL-12. Accordingly, STAT1 is important for signal transduction in natural killer (NK) cells. NK cells are granular lymphocytes and build the front line against virally infected and malignant cells. Loss of STAT1 is associated with a pronounced impairment of NK cell maturation, cytotoxicity and tumor surveillance.^1,2^

In the canonical JAK/STAT signaling cascade binding of cytokines to the respective receptor triggers Janus kinases (JAKs) to phosphorylate STAT1 on tyrosine (Y)701 that allows the formation of active STAT1 dimers. These translocate to the nucleus and bind to stimulus-specific DNA response elements to initiate or repress target gene transcription. Mutation of STAT1-Y701 to phenylalanine (F) precludes phosphorylation and the formation of nuclear translocation-competent STAT1 dimers, thereby abolishing the transcription of typical STAT1 target genes.^3^ Cytokine stimulation induces the successive phosphorylation of STAT1 on Y701 and serine (S)727.^4^ Interestingly, primary NK cells display a pronounced constitutive phosphorylation on S727 that restrains the cytotoxic capacity.^1^ Compared to wild-type, NK cells with mutated STAT1-S727A protein show enhanced cytotoxicity and tumor surveillance. As the canonical function of STAT1-S727 phosphorylation is to increase transcriptional activity, negative regulation by S727-phosphorylated STAT1 (STAT1-pS727) presents a novel aspect of the protein's biology that is mechanistically not understood. As the negative regulation by STAT1-pS727 was observed in absence of discernible pY701, the possibility of STAT1 activity not requiring tyrosine phosphorylation was raised.

To unravel the non-canonical function of STAT1 in NK cells, we utilized *Stat1-Y701F* knock-in mice ^5^ lacking the ability to generate STAT1-pY701 protein. We report that the severely impaired NK cell cytotoxicity of *Stat1*-deficient animals is partially rescued in *Stat1-Y701F* mice. Consistent with its ability to confer NK cytotoxicity STAT1-Y701F partially restored NK cell-mediated tumor surveillance. Mass spectrometry analysis of NK cells expressing a doxycycline-regulated, FLAG-tagged STAT1 (*Stat1*^*ind*^) ^6^ revealed that incubation with target cells causes STAT1 to interact with a plethora of proteins that are important for cell junction formation and found in membrane-bound vesicles. Immunofluorescent staining of primary NK cells uncovered that a large proportion of STAT1 is present at the target cell interface.

## Results

### STAT1-Y701F partially restores NK cell cytotoxicity

**Figure 1.**
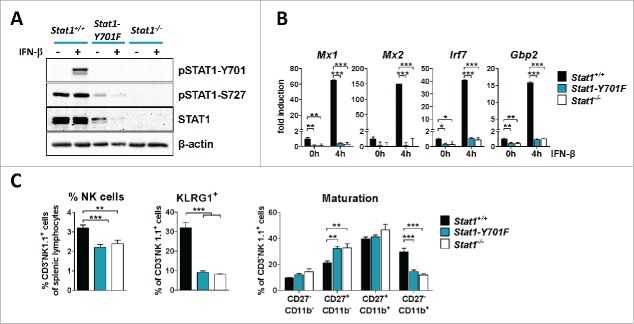
Signaling and maturation of *Stat1-Y701F* NK cells is similar to *Stat1*^*−/−*^ NK cells. (A) Western blot shows STAT1 protein expression and phosphorylation at Y701 and S727 in freshly purified splenic NK cells *ex vivo* and 30 min after treatment with IFN-β. β-actin served as loading control. (B) mRNA expression of *Mx1, Mx2, Irf7* and *Gbp2* was measured by RT-PCR in LAK cells derived from wild-type, *Stat1-Y701F* and *Stat1*^*−/−*^ animals under standard culturing conditions and after IFN-β stimulation for 4 h (n = 3, ***p < 0.001; one-way ANOVA and Tukey's post test). The graphs are representative of two independent LAK cell preparations; all values were normalized to untreated wild-type LAK cells. (C) Flow cytometric analysis of NK cell numbers and NK cell maturation. The panel on the left indicates NK cell fractions among splenic lymphocytes in wild-type, *Stat1-Y701F* and *Stat1*^*−/−*^ mice (n = 12). Middle panel: frequencies of KLRG1^+^ cells (n = 8). Right panel: frequencies of NK subpopulations dissected by CD27/CD11b expression (n = 8). Bar graphs represent mean ± SEM; **p < 0.01, ***p < 0.001; one-way ANOVA and Tukey's post test.

**Figure 2.**
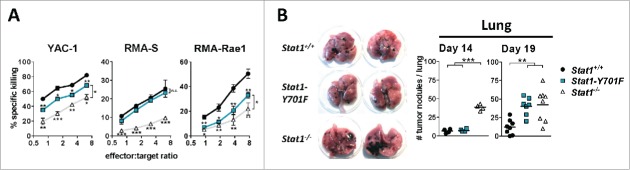
*Stat1-Y701F* NK cells display enhanced cytotoxicity compared to *Stat1*^*−/−*^ NK cells. (A) FACS-based 4 h *in vitro* cytotoxicity assays comparing cytotoxic activities of wild-type, *Stat1-Y701F* and *Stat1*^−/−^ NK against YAC-1, RMA-S and RMA-Rae1 target cells (n = 3 technical replicates; error bars represent SD; ***p < 0.001, **p < 0.01, ***p < 0.001; one-way ANOVA and Tukey's post test of one representative experiment out of three). (B) Pulmonary tumor formation after intravenous injection of 5 × 10^4^ B16F10 melanoma cells into wild-type, *Stat1-Y701F* and *Stat1*^*−/−*^ mice at day 14 (n = 4) and 19 (n ≥ 7 ). **p < 0.01, ***p < 0.001; one-way ANOVA and Tukey's post test. The left panel shows two representative lungs per genotype 14 d after B16F10 inoculation.

To investigate non-canonical functions of STAT1 in NK cells we generated *Stat1-Y701F* knock-in mice.^5^ *Ex vivo* analysis of primary NK cells confirmed the lack of STAT1-Y701 phosphorylation (Fig. 1A) and of transcriptional activation of typical target genes, *i.e. Mx1, Mx2, Irf7* and *Gbp2* (Fig. 1B) upon type I IFN stimulation. Expression of the *Stat1* gene is strongly reduced in cells expressing STAT1-Y701F, owing to the lack of a phosphotyrosine-dependent tonic signal. Despite the drastically reduced STAT1 protein levels in *Stat1-Y701F* NK cells *ex vivo* (Fig. 1A), constitutive phosphorylation on STAT1-S727 was clearly detectable (Fig. 1A), in line with previous observations.^1^ Assessment by flow cytometry demonstrated that the number and maturation of splenic NK cells was impaired in *Stat1-Y701F* mice, comparably to *Stat1*^*−/−*^ NK cells (Fig. 1C). In contrast, we discovered a substantial difference between *Stat1*^*−/−*^ and *Stat1-Y701F* NK cells in their ability to kill tumor target cells. NK cell cytotoxicity was partially restored in *Stat1-Y701F* NK cells in *in vitro* assays upon IL-2 expansion (Fig. 2A and S2A). Noteworthy, we found that *in vitro* cultivation in IL-2 for 5 d enhanced STAT1-Y701F expression levels (Fig. S1). Most importantly the differences in cytotoxicity were not restricted to the *in vitro* situation but also extended to NK cell-dependent tumor surveillance *in vivo*. Upon intravenous injection of B16F10 melanoma cells, *Stat1-Y701F* mice developed only few pulmonary tumor nodules by day 14, whereas *Stat1*^*−/−*^ mice already showed pronounced signs of tumor burden. Tumor development was significantly delayed in *Stat1-Y701F* mice and only at day 19 post injection tumor nodules were clearly visible (Fig. 2B). A similar picture was observed in the liver; whereas *Stat1*^*−/−*^ mice showed clear signs of liver metastasis at day 14 and day 19, this was observed to a lesser degree in *Stat1-Y701F* mice indicating that the effects are not specific for the lung (Fig. S2). This led us to conclude that NK cell-mediated cytotoxicity and tumor surveillance is partially rescued in *Stat1-Y701F* mice.

### Rescue of NK cell cytotoxicity in Stat1-Y701F mice in spite of mostly unaltered transcriptome

**Figure 3.**
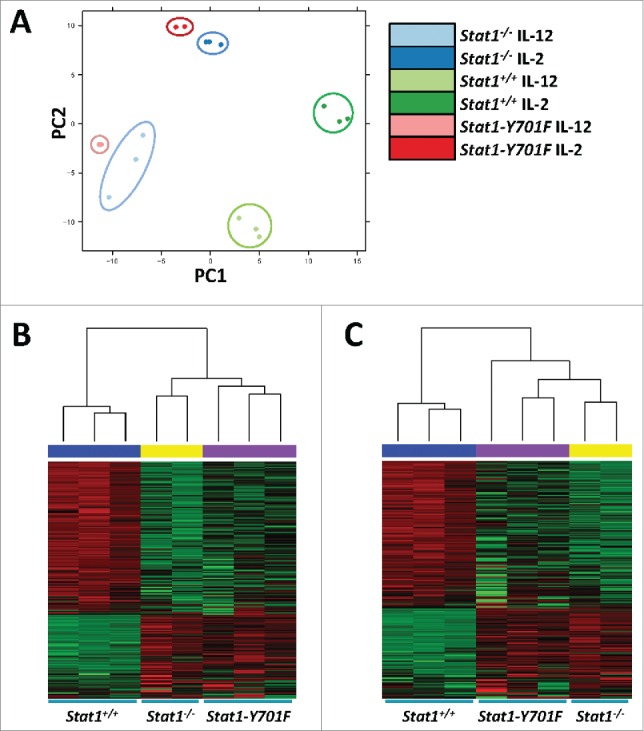
Comparison of RNA-Seq expression signatures. (A) Principal components analysis (PCA) plot of *Stat1*^*+/+*^ (green), *Stat1*^*−/−*^ (blue), and *Stat1-Y701F* (red) samples after IL-12 (light color) and IL-2 (dark color) treatment. PCA is based on 500 genes, which show the highest expression variance between all samples. The first principal component (PC1) separates wild-type from mutated samples. The second principal component (PC2) separates IL-2 from IL-12 treated samples. (B–C) Heat map showing 200 genes with the highest expression variance in (B) IL-2 and (C) IL-12 treated samples. *Stat1*^*+/+*^, *Stat1*^*−/−*^, and *Stat1-Y701F* samples are labeled with blue, yellow and purple bars respectively. Dendrograms on top of the heat map indicate the relative similarity of the expression signatures between each sample. Expression signatures of mutated samples show much higher relative similarity to each other than to wild-type samples.

We next wondered whether a distinct so far unrecognized transcriptional response may be induced in NK cells in the presence of *Stat1-Y701F* that may explain the rescue of NK cell-dependent cytotoxicity and tumor surveillance. To obtain a complete picture of transcriptional changes occurring in a STAT1-dependent manner we performed RNA-seq analysis in *Stat1*^*−/−*^, *Stat1-Y701F* and wild-type NK cells upon stimulation with IL-2 and IL-12. Our efforts are summarized in Fig. 3. In line with the established role of STAT1-pY701 as prerequisite for transcriptional activity, we failed to see any hint for substantial target gene transcription in *Stat1*^*−/−*^ or *Stat1-Y701F* NK cells. When comparing alterations in *Stat1*^*−/−*^ to *Stat1-Y701F* NK cells we obtained a list of seven genes that were significantly altered (either >2-fold upregulation or < 0.5 downregulation; *p* value < 0.01), among which *Stat1* itself served as a positive control (Table S1). Current knowledge and published literature could not provide any obvious link between the transcriptomic alterations in *Stat1-Y701F* NK cells (including changes in *CamK2b, FAM20c* or *CD59a* expression) and the rescue of NK cell cytotoxicity.

### STAT1 interacts with proteins involved in cell junction formation and is associated with membrane or membrane-bound vesicles in NK cells

**Figure 4.**
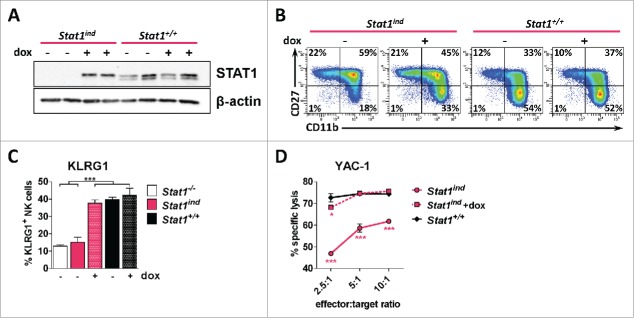
The induced expression of STAT1α in *Stat1*^*ind*^ mice rescues NK cell maturation and cytotoxcity. (A) Splenic NK cells were MACS-purified from *Stat1*^*ind*^ or wild-type mice, which were either untreated (–) or treated with 0.2 mg/mL doxycycline *via* drinking water for 10 d (+) and the expression of STAT1 was analyzed by protein gel blotting. β-actin served as loading control. (B–C) *Stat1*^*ind*^ or wild-type mice were treated with 0.2 mg/mL doxycycline *via* drinking water for 10 d and the maturation profile of splenic NK cells was assessed by flow cytometry. Untreated wild-type and *Stat1*-deficient *Stat1*^*ind*^ mice served as control. (B) The NK cell maturation profile (as determined by the analysis of CD27/CD11b expression on splenic CD3^−^NK1.1^+^NKp46^+^ cells) was partially rescued by STAT1α re-expression as shown in one representative density plot per group. The summary of all analyzed mice is given in Fig. S3. (C) Restoration of wild-type KLRG1 levels in splenic NK cells (gated on CD3^−^NK1.1^+^NKp46^+^ cells) after re-expression of STAT1α, caused by treatment of *Stat1*^*ind*^ mice with doxycycline for 10 d (n = 5–16; bar graphs represent mean ± SEM; ***p < 0.001; one-way ANOVA and Tukey's post test). (D) FACS-based 4 h *in vitro* cytotoxicity assay of LAK cells against YAC-1 target cells. The reduced cytotoxicity of *Stat1*-deficient *Stat1*^*ind*^ mice could be restored by STAT1α re-expression (after *in vivo* and *in vitro* treatment with doxycycline) (n = 3; error bars represent SD; *p < 0.05; ***p < 0.001; one-way ANOVA and Tukey's post test).

We thus wondered whether STAT1 exerts non-canonical functions in NK cells by forming protein signaling complexes with other proteins independent of its tyrosine phosphorylation to promote cytotoxicity. To study protein complexes of STAT1 in primary NK cells we made use of a knock-in mouse harboring a FLAG-tagged allele of STAT1 (*Stat1*^*ind*^). The use of *Stat1*^*ind*^ mice enables the dose-dependent and time-restricted expression of a FLAG-tagged STAT1 protein (STAT1α^FLAG^) on a *Stat1*-deficient background.^6^ Treatment of *Stat1*^*ind*^ mice for 10 d with 0.2 mg/mL doxycycline via drinking water induced the expression of STAT1 in NK cells comparable to wild-type levels (Fig. 4A). The expression of STAT1α largely rescued the impaired maturation of *Stat1*^*−/−*^ NK cells as assessed by flow cytometric analysis of CD27/CD11b on splenic NK cells (Fig. 4B and S3). The partial normalization of CD27/CD11b expression was paralleled by an increase of KLRG1^+^ NK cells comparable to levels observed in wild-type mice (Fig. 4C). The phenotypic rescue induced by STAT1α expression was accompanied by a gain of function: The *in vitro* cytotoxicity of STAT1 re-expressing NK cells against YAC-1 target cells was comparable to wild-type (Fig. 4D). The fact that the maturation and cytotoxic function of *Stat1*-deficient NK cells are rescued by doxycycline-induced STAT1α^FLAG^ expression to wild-type levels allowed us to proceed with proteomic studies.

**Figure 5.**
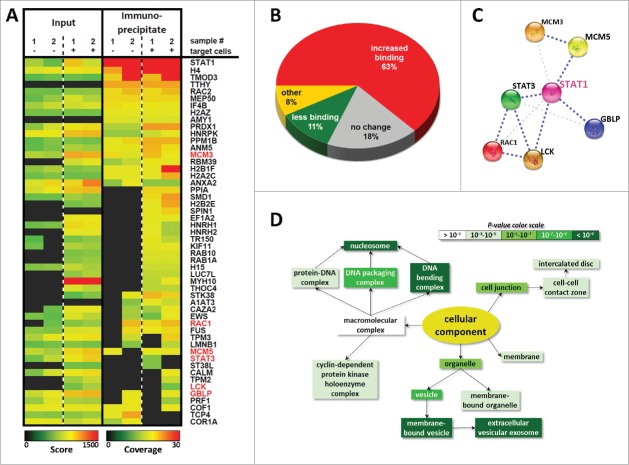
The STAT1α-interactome in primary murine NK cells in homeostasis and upon killing. (A) Doxycycline-treated *Stat1*^*ind*^ LAK cells were used for the IP of STAT1α^FLAG^. The LAK cells either remained untreated (–) or were co-incubated with human Jurkat cells (+) for 30 min. The input controls and immunoprecipitates were investigated by mass spectrometry (n = 2) and an excerpt of interacting proteins is depicted as heat map (for the entire data set please refer to Fig. S5). Data are also available via ProteomeXchange with identifier PXD002206 (project accession: PXD002206; project DOI: 10.6019/PXD002206). In the heatmap the score of the input control and the coverage of the immunoprecipitate is depicted and the scale is given below. (B) A total of 153 STAT1α-interacting proteins were detected in murine NK cells. After co-incubation of the *Stat1*^*ind*^ LAK cells with Jurkat target cells 63% of STAT1α-interaction partners displayed increased interactivity, 11% showed reduced binding and 18% remained unchanged. (C) Mass spectrometry analysis confirmed well-known STAT1-interacting proteins according to the String database (http://string-db.org/). (D) GORILLA analysis (http://cbl-gorilla.cs.technion.ac.il/) of “cellular components” corroborated that STAT1 interacts with DNA and other macromolecular complexes in the nucleus and additionally revealed that STAT1 binds to proteins, which are associated to cell junction, membrane and membrane-bound vesicles with high significance.

To look for binding partners of STAT1 in primary NK cells we performed co-immunoprecipitation (Co-IP) studies of STAT1α^FLAG^ in primary murine *Stat1*^*ind*^ NK cells either unstimulated or co-incubated with human Jurkat cells. The use of human target cells and murine NK cells allows minimizing the rate of false-positive hits as any human protein can clearly be assigned to the tumor cell. Jurkat cells are efficiently killed by mouse NK cells (Fig. S5). An optimized IP protocol was established (Fig. S4), which allowed the precipitation of STAT1α^FLAG^ complexes followed by mass spectrometry analysis (n = 2 biological replicates). This approach detected a total number of 3,581 proteins in murine NK cells, approximately 2% of which were excluded from further validation as they belonged to the families of keratins, immunoglobulins, ribosomal and heat shock proteins and are frequently found as contaminants. Notably, 4.3% of the proteins detected in murine NK cells were found in the precipitated fractions indicating binding to STAT1α (Fig. 5A and S5). Out of the 153 STAT1-binding proteins 18% were found to be constitutively associated to STAT1, 63% were increasingly bound upon target cell co-incubation, 11% were partially dissociated from STAT1 upon target cell co-incubation and 8% were not assignable to any of these groups (Fig. 5B). Fig. 5A shows a short list of STAT1-binding proteins obtained in the mass spectrometry analysis (see Fig. S5 for the entire data set or visit ProteomeXchange: identifier PXD002206). Proteins highlighted in red have been described as STAT1 interaction partners and verified the successful pulldown and specificity of the experiment. Fig. 5C summarizes these well-known STAT1 interaction partners according to the String database (http://string-db.org/). Gene ontology enrichment analysis and visualization tool (GORILLA, http://cbl-gorilla.cs.technion.ac.il/) was used to search for enriched GO terms in our STAT1-interaction partner list compared to the background list of the overall proteome in the input sample. As expected the analysis revealed that STAT1α interacts with DNA and other macromolecular complexes in the nucleus. Consistent with our assumption of an extranuclear function however, several previously STAT1-unrelated GO terms were enriched including cell junction, membrane and membrane-bound vesicles (summarized in Fig. 5D).

To validate the mass spectrometry analysis we performed Co-IP of STAT1α^FLAG^-expressing NK cells either unstimulated or co-incubated with target cells followed by western blotting for distinct proteins. Fig. S6 summarizes these efforts and shows the constitutive and direct interaction of STAT1α^FLAG^ with annexin A2 (Fig. S6A) and the short-term interaction with protein phosphatase 1B (PPM1B) and perforin, which is mainly induced upon target cell killing (Fig. S6B).

### In NK cells STAT1 is recruited to site of target cell contact

**Figure 6.**
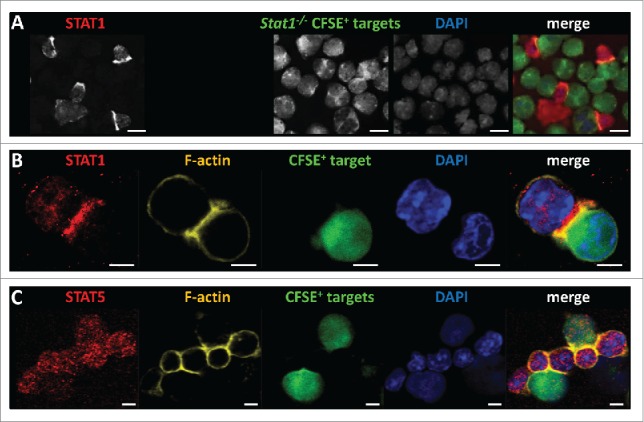
Polarization of STAT1 at the NK-target cell interface. (A) Murine wild-type LAK cells were co-incubated with CFSE-labeled *Stat1*^*−/−*^ v-abl^+^ leukemic cells (green) in a ratio of 1:1 for 30 min prior to the immunofluorescent staining of STAT1 (red). Nuclear staining with DAPI (blue) was included as control. Scale bars: 10 µm. (B) Murine wild-type LAK cells were co-incubated with CFSE-labeled human Jurkat cells (green) for 30 min prior to the immunofluorescent staining of STAT1 (red) and F-actin (yellow). Upon tumor cell challenge STAT1 remained cytoplasmic and was partially found in the NK-immunological synapse, as it co-localized with F-actin. Nuclear staining with DAPI (blue) was included as control. Scale bars: 5 µm. (C) Murine wild-type LAK cells were co-incubated with CFSE-labeled human Jurkat cells (green) for 30 min prior to the immunofluorescent staining of STAT5 (red) and F-actin (yellow). Upon tumor cell challenge STAT5 remained evenly distributed over the NK cell and was not recruited to the NK immunological synpase as it did not co-localize with F-actin. Nuclear staining with DAPI (blue) was included as control. Scale bars: 5 µm.

The proteomic data indicate that STAT1 may contribute to the cytotoxic process by interacting with cell junction and membrane-associated proteins. We thus wanted to investigate whether the proposed non-transcriptional activity of STAT1 in the course of NK cell killing is reflected by changes in the subcellular localization of STAT1. We scrutinized the spatial distribution of STAT1 proteins in wild-type NK cells upon contact with target cells. Primary murine wild-type NK cells were co-incubated for 30 min with leukemic cells followed by immunofluorescent staining. We chose *Stat1*^*−/−*^ leukemic cells as they represent prime NK cell targets due to their low expression levels of MHC class I.^7^ In addition they served as optimal experimental control for STAT1 antibody specificity. The outcome of the experiment was clear-cut: as shown in Fig. 6A the majority of STAT1 proteins was polarized toward the tumor target cell and assembled at the cell–cell interface. There STAT1 co-localized with F-actin (Fig. 6B), which is a well-established marker for the immunological synapse.^8^ During NK cell killing the majority of the STAT1 protein pool remained cytoplasmic with a significant fraction being recruited to the area of the IS; only a minor fraction of STAT1 protein was present in the nucleus. Contrasting STAT1, STAT5 was not polarized toward the target cell and remained evenly distributed all-over the NK cell (Fig. 6C). This led us to conclude that STAT1 exerts a non-canonical transcription-unrelated function at the NK-target cell interface that is not shared by other members of the STAT transcription factor family.

## Discussion

STAT1 is an essential mediator of immunity against microorganisms and tumors. Its vital importance derives from the employment by a number of different cytokine receptors that include IFN receptors and the IL-12 receptor. As both type I IFN and IL-12 are critical regulators of NK cell activity the impairment of cytotoxicity in *Stat1*^*−/−*^ mice meets the expectations. Surprisingly, however, STAT1 affects NK cell biology beyond its canonical role as an activator of immediate transcriptional responses. This notion emerged from our recent observation that phosphorylation at S727 bestows upon STAT1 the ability to restrict NK activity in the absence of detectable phosphorylation at Y701.^1^ Following up on this finding, we now show that STAT1-Y701F partially rescues the defect of *Stat1*^*−/−*^ NK cells both *in vitro* and *in vivo*. The partial restoration of NK cell cytotoxicity occurred despite strongly reduced protein amounts underscoring its importance. In line with a non-nuclear and non-canonical activity of STAT1 in NK cells, the protein localized to the NK cell/target cell interface and co-purified with membrane-associated and vesicular proteins.

Unphosphorylated STAT1 (U-STAT1) proteins predominantly localize to the cytoplasm as inactive homodimers.^9–11^ In spite of this, nuclear and transcriptional activity has been assigned to U-STAT1.^12–15^ This pY701-independent transcriptional function occurs upon upregulation of STAT1 through canonical signaling. As this feed-forward loop is impaired or even absent in *Stat1-Y701F* cells, insufficient STAT1 amounts are established for significant transcriptional U-STAT responses. Thus, the U-STAT1 pathway as originally described by *Stark and colleagues* is unlikely to rescue NK activity. Strong support for this argument stems from our RNA-seq data that showed only minor differences in transcriptional responses in *Stat1-Y701F* NK cells compared to *Stat1*^*−/−*^ controls. Seven genes were found significantly altered including *Stat1*, which served as control. Neither the altered expression of *CD59a*, nor of *Fam20c* or *CamK2b* provided a satisfactory explanation for the rescue of cytotoxicity. Fam20C is a kinase phosphorylating secreted proteins in a rather non-specific manner accounting for the majority of the secreted phospho-proteome.^16^ *CD59a*^*−/−*^ mice have been generated and show a mild hemolytic phenotype and an increased sensitivity to complement-dependent lysis.^17^ CaMKIIb is a serine-threonine kinase associated predominantly with neuronal functions.^18^ We have currently no insights in the function of the so far solely RIKEN-annotated genes.

Despite the fact that we cannot formally rule out a transcriptional contribution to the observed phenotype we propose the existence of an unusual non-canonical function of STAT1 at the NK cell interface with its target cell. Our data provide strong evidence that the presence of STAT1 is required at the immunological interface to enable NK cell cytotoxicity—thereby explaining the rescue that we observed upon expression of *Stat1-Y701* in a *Stat1*-deficient background.

Recent years have seen the emergence of several phosphotyrosine-independent STAT functions in the cytoplasm, mitochondria or the cell nucleus.^19–23^ Important in the context of our results, inactive Drosophila STAT was found associated with the apical membrane of epithelial cells in proximity to protein complexes regulating cell polarity.^24^ In mammalian cells a fraction of U-STAT3 is in contact with the plasma membrane and with signaling endosomes.^25^ These studies emphasize a role of STATs in membrane trafficking and organelle-associated signaling. At present, we cannot mechanistically explain the contribution of the U-STAT1 fraction at the target cell interface to NK cytotoxicity.

However, the presence of the protein in an IS is not unprecedented. The differentiation of Th precursors (Thp) to the Th1 lineage requires signaling via the T cell receptor (TCR) and the IFNγ receptor (IFNGR). Activation of Thp cells induces the co-recruitment of TCR and IFNGR to the synapse and drives their differentiation into Th1 effector cells.^26^ STAT1 is co-recruited to the Thp-IS, binds to the IFNγ receptor at the cell membrane and translocates to the nucleus.^27^ Similar to NK cells, Thp STAT1 is constitutively phosphorylated on S727 in this situation, but remains Y701 unphosphorylated. Our purification of STAT1-associated NK cell proteins has not yielded any evidence for an association with the IFNγ or other cytokine receptors, possibly due to the transient nature of the interaction, or due to limitations of the methodology. Our protocol was not optimized for the purification of membrane-bound proteins. In this light the high significance of STAT1 interaction with membrane proteins as revealed by GORILLA analysis is striking and similarities between STAT1 function in the IS of NK and Thp cells require further exploration.

In conclusion we provide the first evidence of U-STAT function in animals carrying a genomic mutation of the critical tyrosine residue. By demonstrating a membrane-associated function of U-STAT1 in NK cells we add an important new aspect to the complex biology and diverse employment of STATs in cell signaling and transcriptional regulation.

## Material and methods

### Mice

Gender- and age-matched 6–12 week old C57BL/6J wild-type (*Stat1*^*+/+*^), B6;129P2-*Stat1*^*tm1Dlv*^/J (*Stat1*^*−/−*^),^28^ B6;129S6Sv-*Stat1*^*tm1(Y701F)*^ (*Stat1-Y701F*) ^5^ and B6N;129P2-*Stat1*^*tm1Dlv*^*, Gt(RO-SA)26Sor*^tm1(rtTA*M2)Jae^*, Col1a1*^*tm1(tetO-Stat1)Biat*^ (referred to as *Stat1*^*ind*^mice)^6^ mice were used and housed under specific pathogen-free conditions according to FELASA guidelines. All experiments were approved by the Ethics and Animal Welfare Committee of the University of Veterinary Medicine Vienna and conform to the guidelines of the national authority (the Austrian Federal Ministry for Science and Research). *Stat1*^*ind*^ and control mice were fed with doxycycline hyclate (dox; Sigma) at concentrations of 0.2–0.5 mg/mL supplemented with 5–10 mg/mL sucrose via drinking water for 7–10 d.

### Cell culture

NK cells were isolated from splenocytes using the MACS NK cell separation kit (anti-DX5 microbeads, Miltenyi Biotec). Murine primary NK cells, YAC-1, RMA-S, RMA-Rae1, *Stat1*^*−/−*^ v-abl^+^ leukemic and B16F10 melanoma cells were cultivated as previously described.^1^ Human Jurkat cells were maintained in RPMI-1640 medium containing L-glutamine (PAA), 10% fetal calf serum (FCS, PAA), 50 µM 2-mercaptoethanol (Sigma-Aldrich), 100 U/mL penicillin and 100 µg/mL streptomycin (Life Technologies). Lymphokine-activated killer (LAK) cells were maintained in 5,000 U/mL rhIL-2 (Proleukin, Novartis) for 5–7 d and treated with 250 U/mL rmIFN-β (PBL) for 4 h prior to gene analysis. *Stat1*^*ind*^ LAK cells were continuously cultivated in the presence of 50 ng/mL doxycycline.

### Co-immunoprecipitation (Co-IP) and Western Blotting

For Co-IP experiments 3 × 10^6^ NK cells were harvested and stimulated for 10 min with 100 U/mL rmIFN-β (Merck Millipore) or co-incubated for 30 min with 3 × 10^6^ Jurkat cells before the lysis in 200 µL IP lysis buffer. The composition of the optimal buffer to precipitate STAT1 complexes was determined experimentally (Fig. S4): 50 mM HEPES pH 7.5, 0.1% Tween-20, 150 mM NaCl, 1 mM EDTA, 10 mM β-glycerophosphate, 1 µM PMSF, 1 mM NaF, 500 nM Na_3_O_4_V and complete Protease Inhibitor Cocktail Tablets (Roche). STAT1α^FLAG^ was precipitated with the use of the ANTI-FLAG M2 Affinity Gel (Sigma-Aldrich). As controls served 5% of the input (whole cell lysate) and 5% of the bead-supernatant (not bound to the beads). The proteins bound to the αFLAG-beads were eluted by cooking for 10 min in 4x Laemmli-buffer freshly complemented with β-mercaptoethanol.

Western Blotting was performed as described previously.^1^ In brief, proteins were separated by SDS/PAGE and transferred to nitrocellulose membranes (Whatman® Protran®). After blocking with 5% BSA, membranes were probed with antibodies against STAT1 C-term,^29^ STAT1 (sc-592), β-actin (sc-69879), α-tubulin (sc-32293) (Santa Cruz Technology), STAT2 (CS#4597), pSTAT1-Y701 (CS#9171), pSTAT1-S727 (CS#9177) and perforin (CS#3693) (Cell Signaling), annexin A2 (610068) (BD Biosciences) and PPM1B (ab70804) (abcam). Immunoreactive bands were visualized by chemiluminescent detection (Clarity Western ECL substrate, Bio-Rad) by the ChemiDoc MP Imaging System (Bio-Rad Laboratories, CA, USA).

### Mass spectrometry

In-solution digestion: If not stated otherwise, all reagents were obtained from Sigma. The total lot of beads obtained per IP experiment was used for proteolytic digestion. Beads were washed with 50 mM ammonium bicarbonate (ABC buffer) on top of conditioned 3 kD MWCO filters (Pall Austria Filter GmbH) by centrifugation at 14,000 g for 15 min. After reduction with 200 µL of dithiothreitol solution (5 mg/mL dissolved in 8 M guanidinium hydrochloride in ABC buffer at pH 8) and alkylation with 200 µL of iodacetamide solution (10 mg/mL in 8 M guanidinium hydrochloride in ABC buffer), proteins were digested 18 h at 37°C using 10 µL trypsin solution (0.1 µg/µL). Clean up of peptide samples were performed using C-18 spin columns (Pierce, Thermo Scientific). Finally, the peptide samples were dried and stored at −20°C until MS analyses. For shotgun-analyses, dried samples were reconstituted in 5 µL 30% formic acid (FA) containing 10 fmol each of four synthetic standard peptides and diluted with 40 µL mobile phase A (98% H_2_O, 2% ACN, 0.1% FA). Synthetic peptides [Glu1-Fribrinopeptide B – EGVNDNEEGFFSAR; M28 – TTPAVLDSDGSYFLYSK; HK0 – VLETKSLYVR; HK1 – VLETK(ϵ-AC)SLYVR] were obtained from Sigma and Peptide Specialty Laboratories GmbH and spiked in each sample as internal quality control for monitoring LC-MS-system stability.

Shotgun LC-MS analysis: 10 µL of the peptide samples were injected into a Dionex Ultimate 3000 nano LC-system coupled to a QExactive orbitrap mass spectrometer equipped with a nanospray ion source (Thermo Fisher Scientific). All samples were analyzed in technical duplicates. For pre-concentration, peptides were loaded on a 2 cm × 75 µm C18 Pepmap100 pre-column (Thermo Fisher Scientific) at a flow rate of 10 µL/min using mobile phase A. Elution from the pre-column to a 50 cm × 75 µm Pepmap100 analytical column (Thermo Fisher Scientific) and separation was achieved at a flow rate of 300 nL/min using a gradient of 8% to 40% mobile phase B (80% ACN, 20% H_2_O, 0.1% FA) over 95 min. For mass spectrometric detection, MS scans in the range from m/z 400–1400 at a resolution of 70,000 (at m/z = 200) were performed. MS/MS scans of the eight most abundant ions were achieved through HCD fragmentation at 30% normalized collision energy and analyzed in the orbitrap at a resolution of 17,500 (at m/z = 200).

Shotgun LC-MS data analysis: ProteomeDiscoverer 1.4 (Thermo Fisher Scientific, Austria) running Mascot 2.4 (Matrix Science, UK) was used for protein identification and qualitative data analysis. Protein identification was achieved searching against the SwissProt/UniprotKB Database (version 052014 including only reviewed proteins with 20,263 entries) allowing a mass tolerance of 5 ppm for MS spectra and 20 ppm for MS/MS spectra as well as a maximum of two missed cleavages. Furthermore, search criteria included carbamidomethylation on cysteins as fixed modification and methionine oxidation as well as N-terminal protein acetylation as variable modifications. A list of lab-characteristic contaminants including various keratins was excluded manually. The FDR for peptide spectrum matches was set to < 0.01, in addition the Mascot significance threshold was set to 0.01.

The mass spectrometry proteomics data have been deposited to the ProteomeXchange Consortium^30^ via the PRIDE partner repository with the dataset identifier PXD002206 and 10.6019/PXD002206.^31^

Mass spectrometry data representation: The raw data was combined in a single Excel file and filtered for proteins found in the immunoprecipitate, i.e. the coverage in the IP > 0 in at least one of the four samples. Those hits (n = 153) were assigned to one of the following four groups: (i) proteins that are constitutively associated to STAT1α and found at comparable levels (coverage in the IP is constant) in unstimulated and target-stimulated NK cells (n = 40), (ii) proteins that are increasingly bound to STAT1α upon target cell co-incubation (coverage in the IP of target-stimulated > coverage in the IP of unstimulated; n = 80), (iii) proteins that are lost or less bound to STAT1α upon target cell co-incubation (coverage in the IP of target-stimulated < coverage in the IP of unstimulated; n = 23), and (iv) not assignable to either of these groups (n = 10). Within the four groups the hits were sorted according to the coverage in the IP in decreasing order. A colorimetric heatmap (three-color scale) was generated using the conditional formatting in Excel: green (low values), yellow (set at 75% percentile), red (high values). Zero values were displayed in black. The score of the corresponding input samples was displayed side-by-side for each protein respectively.

### Flow Cytometry

Freshly isolated splenocytes were depleted of erythrocytes and treated for 5 min with purified anti-CD16/32 to avoid non-specific binding prior to staining with fluorescently-labeled antibodies. Splenocytes were stained for 20 min at 4°C with the following antibodies (clones): CD3ϵ (145-2C11), CD11b (M1/70), CD27 (LG.3A10) and KLRG1 (2F1) (BD Biosciences); CD3ϵ (17A2, 145-2C11), NK1.1 (PK136) and NKp46 (29A1.4) (eBioscience). The samples were recorded on a FACSCanto II flow cytometer (BD Biosciences, Heidelberg, Germany) and analyzed with BD FACSDiva software version 7.1.2.

### Semiquantitative real-time PCR

RNA was prepared from MACS-purified and *in vitro* expanded LAK cells using NucleoSpin II® isolation kit (Macherey-Nagel). 200 ng RNA was reversely transcribed by ReverseAid reverse transcriptase (Thermo Scientific). Real-time PCR was performed on a Realplex real-time cycler (Eppendorf, Hamburg, Germany) with GoTaq® qPCR master mix (Promega) according to the manufacturer's instructions. The applied primer sets were as follows (5′3′): *Mx1*: fw GACTACCACTGAGATGACCCAGC, rev ATTTCCTCCCCAAATGTTTTCA; *Mx2*: fw CCAGTTCCTCTCAGTCCCAAGATT, rev TACTGGATGATCAAGGGAACGTGG; *Irf7*: fw ATTTCGGTCGTAGGGATCTGG, rev GCACAGCGGAAGTTGGTCT; *Gbp2*: fw TGCTAAACTTCGGGAACAGG, rev GAGCTTGGCAGAGAGGTTTG. Target gene expression was normalized to *Gapdh* ^1^ and set relative to untreated wild-type LAK cells.

### RNA-seq

Splenic NK cells were isolated using the MACS positive selection kit (DX5, Miltenyi) and cultured with 5000 U/mL rhIL-2 (Proleukin®, Roche) for 5 d. NK cells were sorted for CD3^−^NK1.1^+^ on a FACS AriaIII (BD) and samples were stimulated for 3 h with 5000 U/mL rhIL-2 ± 5 ng/mL rmIL-12 (R&D). RNA was isolated by RNeasy Micro Kit (Qiagen) and RNA-seq has been analyzed based on the GRCm38 V17 mouse genome and gene annotation.

Sequencing and read processing: Paired-end 100 bp reads have been generated with the Illumina TruSeq protocol. On average 34 million read pairs have been generated for each sample. Reads were then aligned to the mouse reference genome (mm10) with the GSNAP aligner version 2012-12-20, which is an accurate splice-junction mapper for RNA-Seq data.

Differential gene expression analysis: To determine gene expression levels, we counted the number of reads for 38,293 mouse genes using HTSeq version 0.5.3 based on the Ensembl gene annotation version 71. We then used EDASeq (Version 1.4.0) to correct for GC-content bias and tested for differential gene expression using DESeq (Version 1.10.1).

Heatmaps and PCA clustering: Plots have been generated based on the 200 and 500 genes, which showed the strongest expression variance between the samples for heat map plot and PCA clustering respectively. Expression variances were calculated for samples which are shown in the plots only (all samples for PCA clustering and eight samples for heat map plots).

### Immunofluorescent staining

Immunofluorescent stainings were performed in poly-D-lysine-coated 8-well Millicell EZ SLIDES (Merck Millipore). 10^6^ wild-type LAK cells were co-incubated in a ratio of 1:1 with CFSE-labeled (2.5 µM; CellTrace CFSE Cell Proliferation Kit, Molecular Probes) murine *Stat1*^*−/−*^ v-abl^+^ leukemic or human Jurkat cells in RPMI complete medium. Cells were washed and fixed after 30 min in 3% para-formaldehyde (20 min) followed by neutralization in 50 mM NH_4_Cl (30 min) and permeabilization with 0.1% TritonX-100 (10 min). After washing with TBS (2 × 30 min) the slides were blocked at 4°C in 3% BSA and 2.5% goat serum (90 min) and incubated with the primary antibodies overnight diluted 1:500 in 3% BSA and 1% goat serum in TBS-T [STAT1 (sc-592) or STAT5 (sc-835)]. To visualize F-actin each well was stained with 1 unit (5 µL) Alexa Fluor 546 Phalloidin (A22283, Life Technologies). After three washing steps the cells were stained with Alexa Fluor 647 goat anti-rabbit IgG antibody (A-21245, Life Technologies) diluted in 3% BSA and 5% goat serum in TBS (120 min). After washing the slides were mounted in VECTASHIELD HardSet Mounting Medium with DAPI (H-1500, Vector Laboratories). Pictures were taken on a ZEISS AxioImager Z1 fluorescence microscope with a 20x objective or a ZEISS LSM 510 META confocal laser scanning microscope with a Plan-Neofluar 40x/1.3 Oil DIC objective (ZEISS, Oberkochen, Germany) and analyzed by the ZEN 2009 software.

### NK cytotoxicity assay

Flow cytometry-based *in vitro* cytotoxicity assays were performed as described previously.^1^

### B16F10 tumor model

Mice were injected *via* tail vein with 5 × 10^4^ B16F10 melanoma cells and sacrificed after 14 or 19 days, respectively. Lung and liver were harvested and captured on camera. Tumor nodules visible on their surface were counted under a microscope.

### Statistical analysis

Statistical analysis was performed using GraphPad Prism® version 5.00 for Windows (GraphPad Software, San Diego, CA, www.graphpad.com). Where ANOVA showed a statistical difference, Tukey's multiple comparison testing was applied. The α level for all tests was set to 0.05 and *p* values were 2-tailed. Statistical analysis is indicated for each experiment (in general: *p < 0.05; **p < 0.01; ***p < 0.001).

## Supplementary Material

KONI_A_1186314_s02.zip
