# Supplementary figures and images for: Novel non-canonical role of STAT1 in Natural Killer cell cytotoxicity
Source: Oncoimmunology. 2016 May 19;5(9):e1186314. doi: 10.1080/2162402X.2016.1186314 (PMC5048756; doi:10.1080/2162402X.2016.1186314)

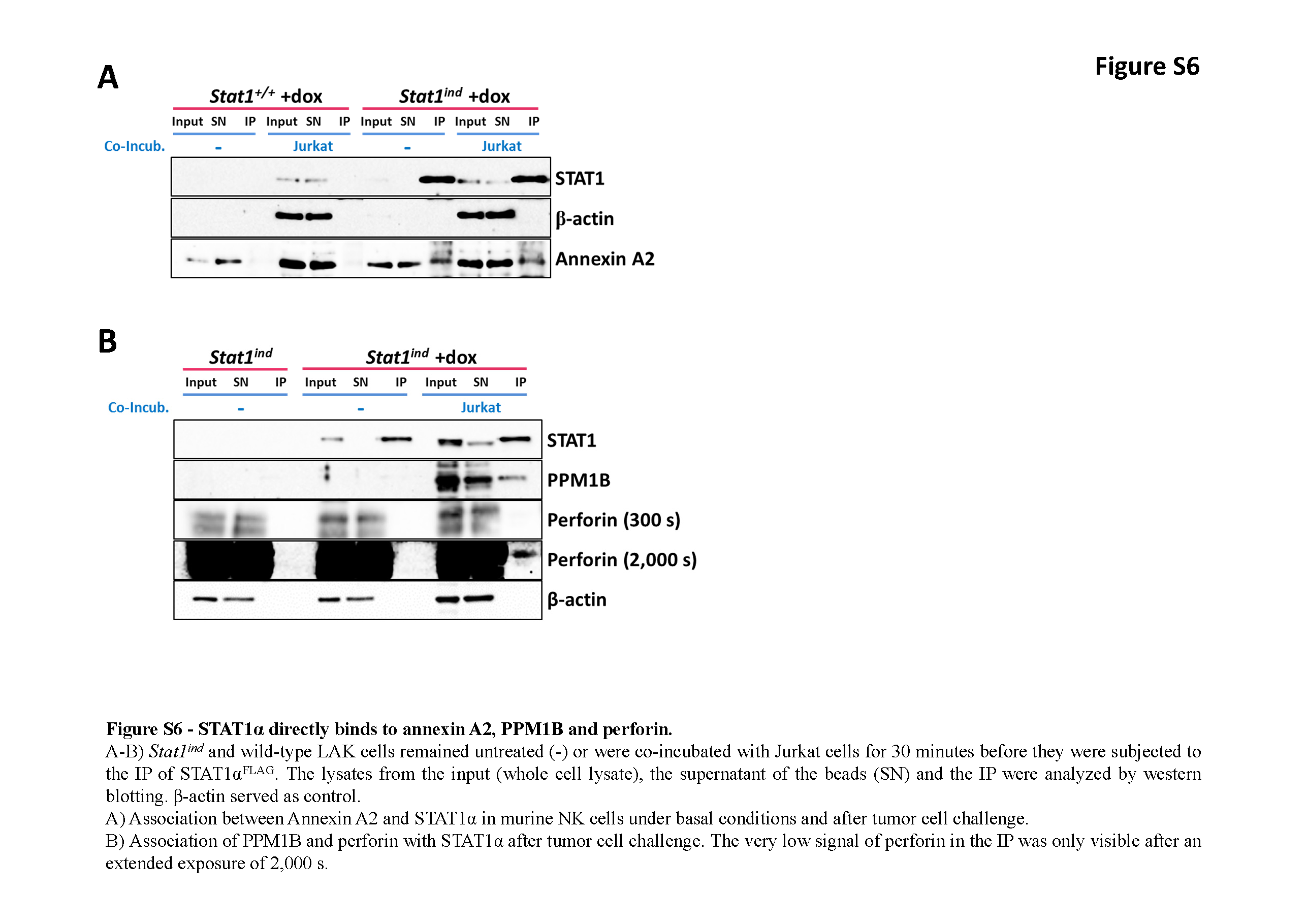

Supplement: KONI_A_1186314_s02.zip [file koni-05-09-1186314-s001.zip › 2015ONCOIMM0671R-f12-z-4c.tif]

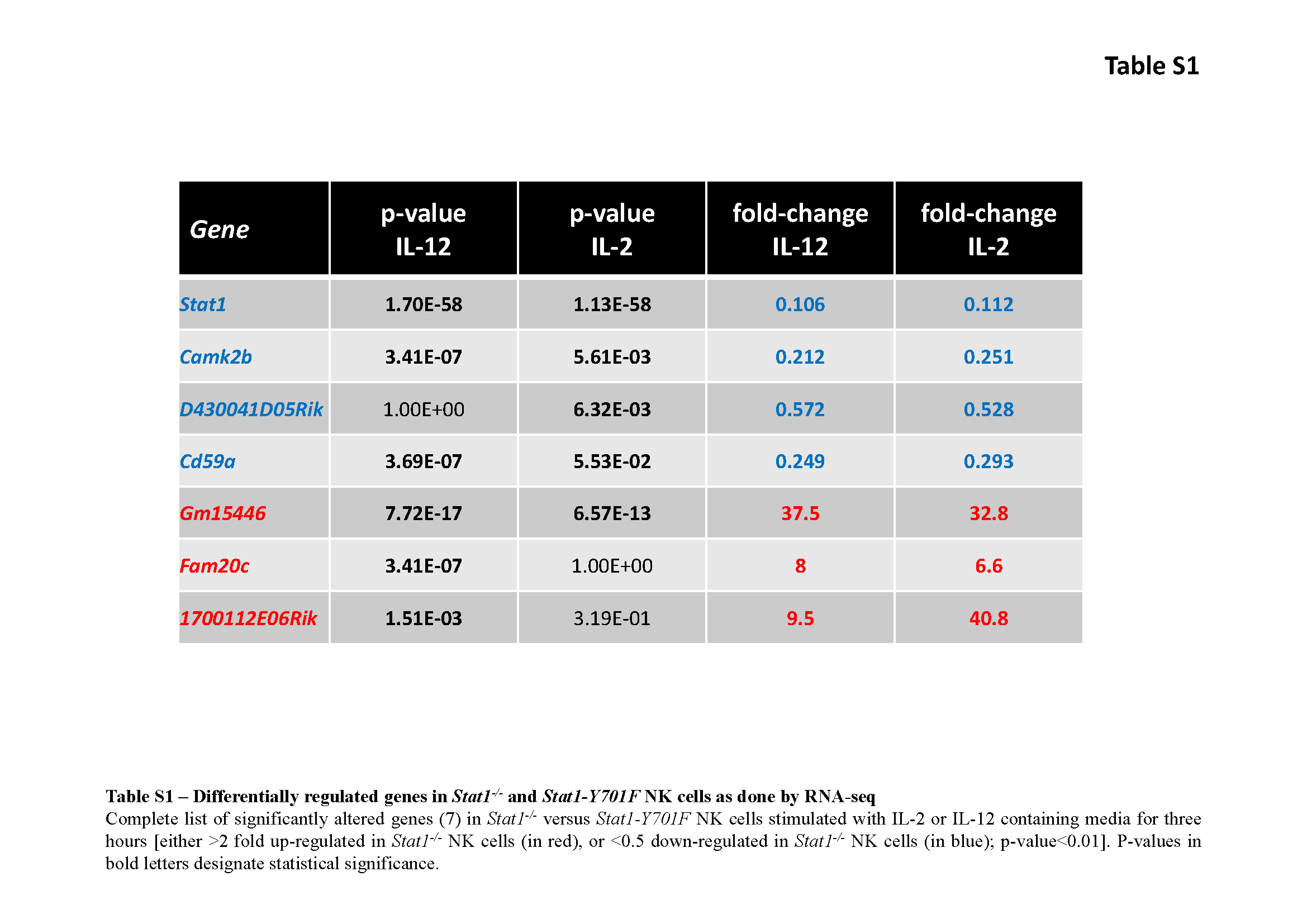

Supplement: KONI_A_1186314_s02.zip [file koni-05-09-1186314-s001.zip › 2015ONCOIMM0671R-file002.tif]

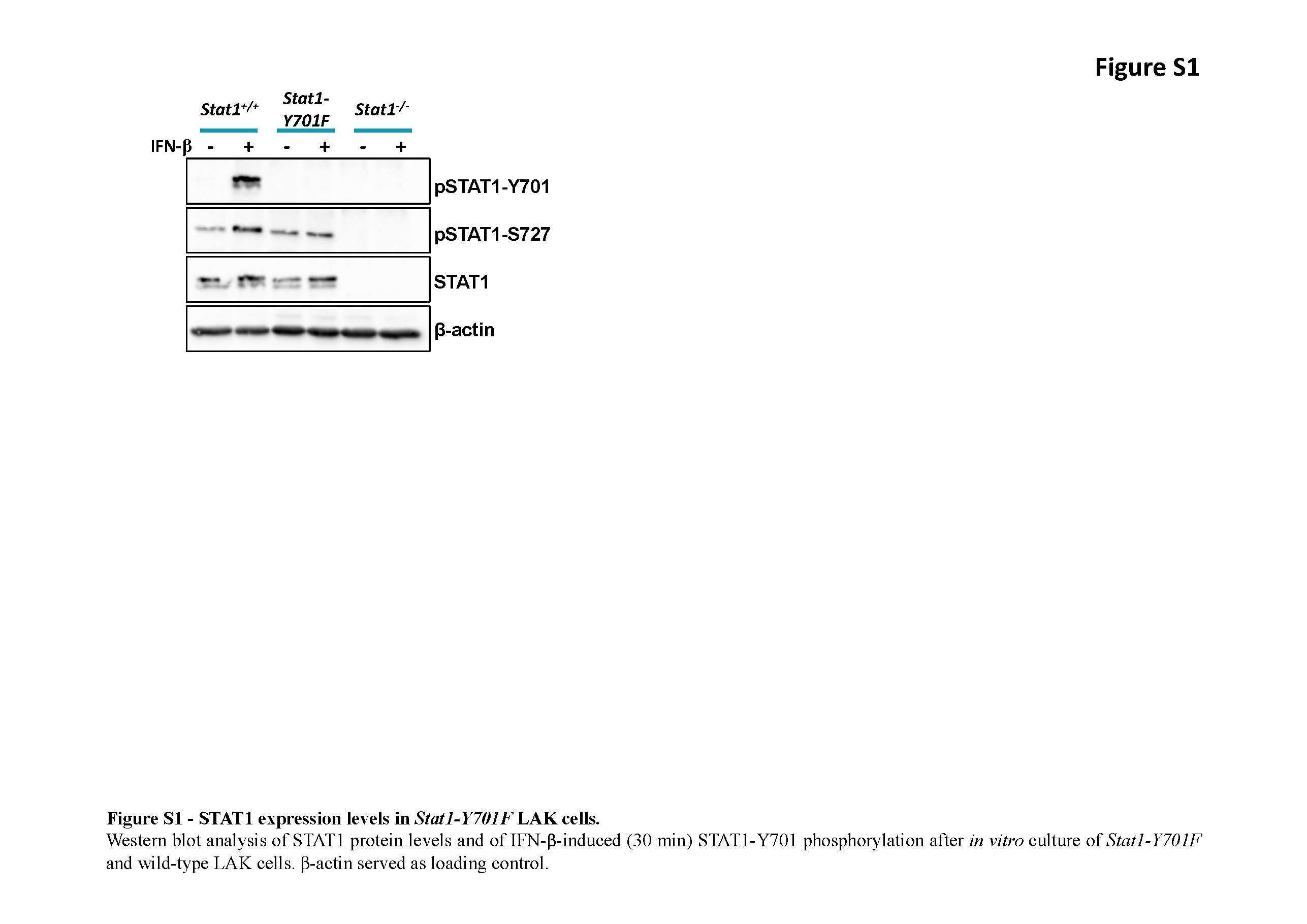

Supplement: KONI_A_1186314_s02.zip [file koni-05-09-1186314-s001.zip › 2015ONCOIMM0671R-f07-z-4c.tif]

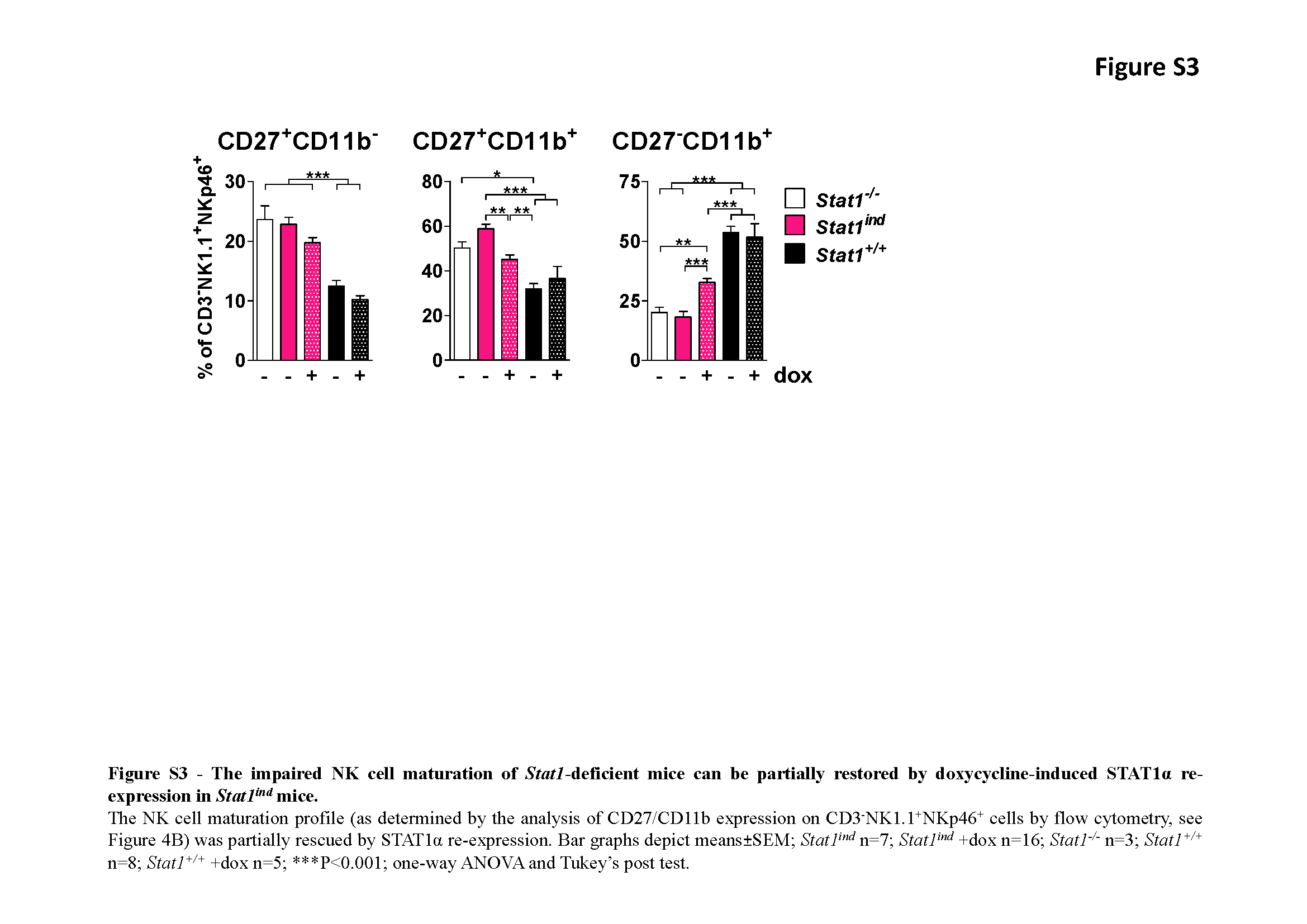

Supplement: KONI_A_1186314_s02.zip [file koni-05-09-1186314-s001.zip › 2015ONCOIMM0671R-f09-z-4c.tif]

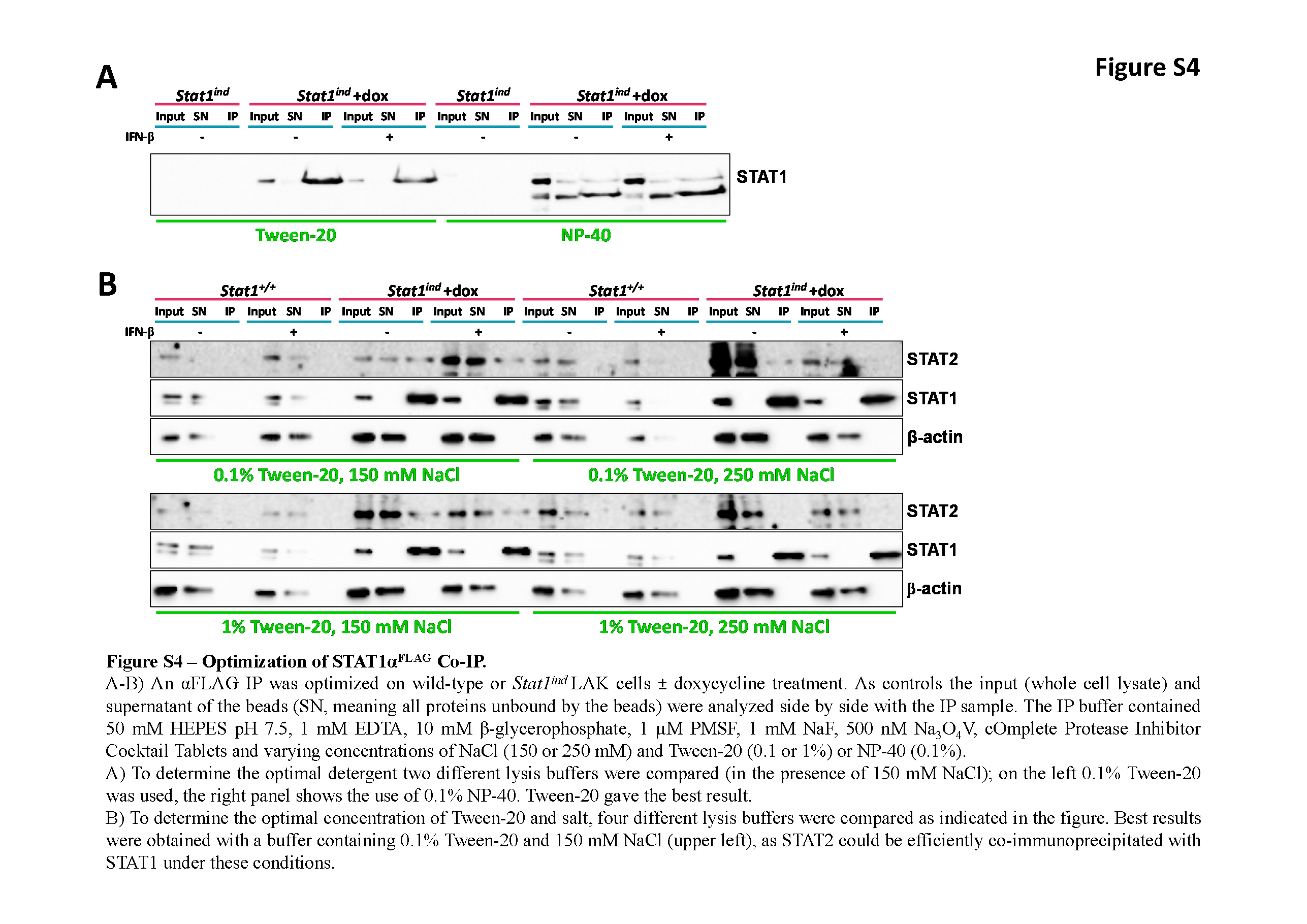

Supplement: KONI_A_1186314_s02.zip [file koni-05-09-1186314-s001.zip › 2015ONCOIMM0671R-f10-z-4c.tif]

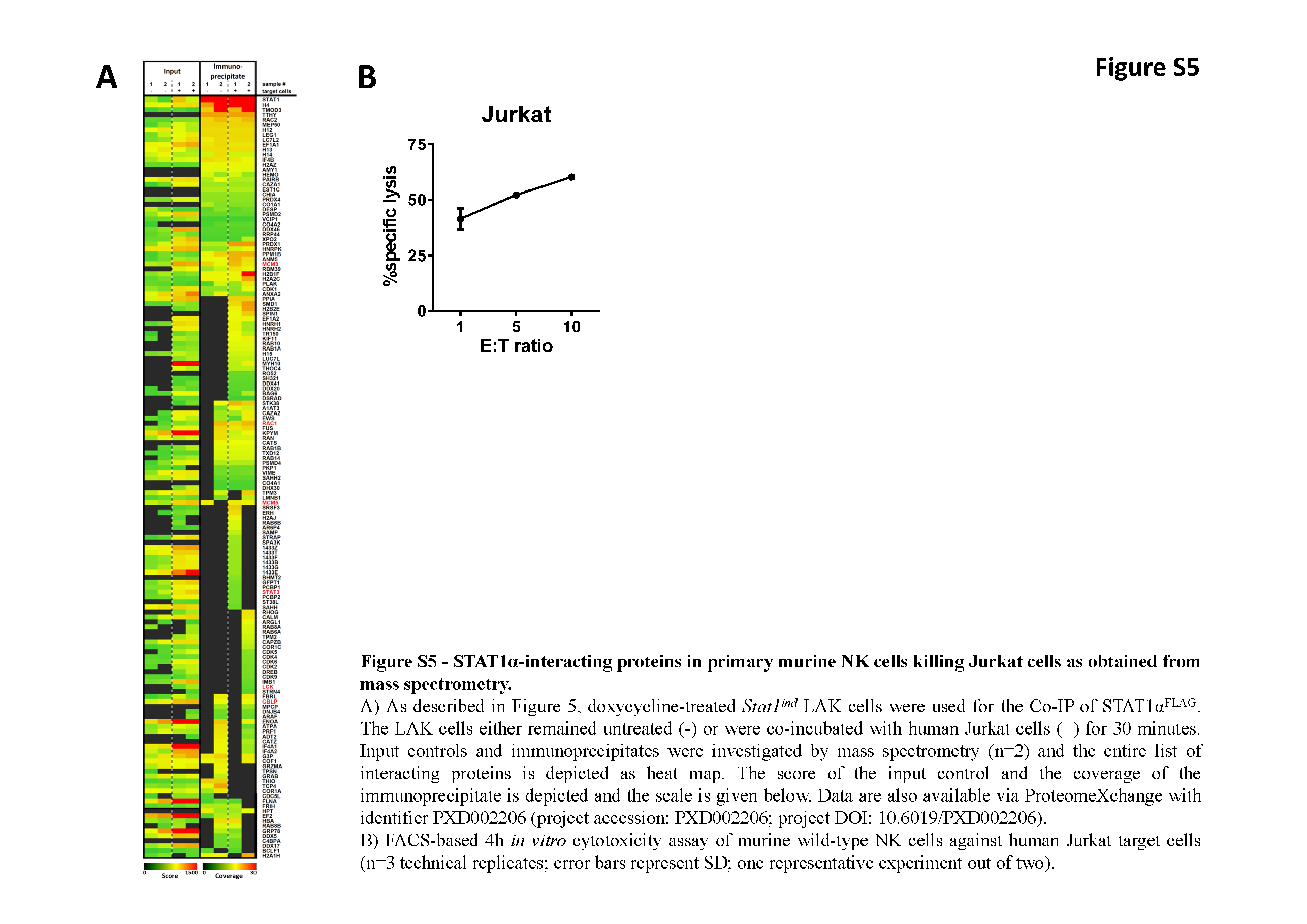

Supplement: KONI_A_1186314_s02.zip [file koni-05-09-1186314-s001.zip › 2015ONCOIMM0671R-f11-z-4c.tif]
